# Artificial intelligence for response prediction and personalisation in radiation oncology

**DOI:** 10.1007/s00066-024-02281-z

**Published:** 2024-08-30

**Authors:** Alex Zwanenburg, Gareth Price, Steffen Löck

**Affiliations:** 1 OncoRay—National Center for Radiation Research in Oncology, Faculty of Medicine and University Hospital Carl Gustav Carus, TUD Dresden University of Technology, Helmholtz-Zentrum Dresden-Rossendorf, Fetscherstr. 74, PF 41, 01307 Dresden, Germany; 2 National Center for Tumor Diseases Dresden (NCT/UCC), Germany:, German Cancer Research Center (DKFZ), Heidelberg, Germany; Faculty of Medicine and University Hospital Carl Gustav Carus, TUD Dresden University of Technology, Dresden, Germany; Helmholtz-Zentrum Dresden-Rossendorf (HZDR), Dresden, Germany; 3 German Cancer Research Center (DKFZ) Heidelberg, Heidelberg, Germany; 4 Division of Cancer Sciences, University of Manchester, Manchester, UK; 5 The Christie NHS Foundation Trust, Manchester, UK; 6 Department of Radiotherapy and Radiation Oncology, Faculty of Medicine and University Hospital Carl Gustav Carus, TUD Dresden University of Technology, Dresden, Germany

**Keywords:** Radiotherapy, Artificial intelligence, Tumour control probability, Treatment response, Normal tissue complication probability

## Abstract

Artificial intelligence (AI) systems may personalise radiotherapy by assessing complex and multifaceted patient data and predicting tumour and normal tissue responses to radiotherapy. Here we describe three distinct generations of AI systems, namely personalised radiotherapy based on pretreatment data, response-driven radiotherapy and dynamically optimised radiotherapy. Finally, we discuss the main challenges in clinical translation of AI systems for radiotherapy personalisation.

## Introduction

Radiotherapy is one of the pillars of cancer treatment [1], using ionising particles or x‑rays to arrest development of the treated tumour, i.e. to establish tumour control. Tumour control tends to improve with increasing radiation dose, although clinical evidence paints a more complex picture [2]. At the same time, radiation also negatively affects healthy tissues. These radiotherapy side effects range from minor to severe transient or chronic disabilities that negatively impact patient wellbeing. Therefore, radiotherapy is often a compromise between achieving good tumour control while limiting severe side effects. Currently, such considerations are based on observations in populations of treated patients with similar diagnoses, and result in a treatment plan with a standard radiation dose. However, both tumour control and healthy tissue response are known to be patient specific. Therefore, there is an ongoing effort to personalise radiotherapy [3].

Sometimes single characteristics or biomarkers strongly affect tumour control or the healthy tissue response. For example, oropharyngeal cancers caused by human papillomavirus type 16 are more radiosensitive and easier to treat than oropharyngeal cancers of different origins [4]. In other cases, there may not be a single biomarker or small set of biomarkers that determines or correlates with tumour control or the healthy tissue response. Instead, these are driven by a complex variety of characteristics.

Artificial intelligence (AI) systems can aid in providing clinical insights based on complex sets of characteristics. In this article, we discuss the use of AI systems to predict radiotherapy response and discuss ongoing research and concepts regarding how such systems might be used for personalised radiotherapy.

## Personalisation before treatment

Prior to treatment, AI systems can personalise treatment by predicting the estimated response of the tumour and healthy tissues to radiotherapy. In essence, such systems extend existing tumour control probability (TCP) and normal tissue complication probability (NTCP) models. TCP models currently inform the prescribed standard fractionation and radiation dose, whereas NTCP models inform dose constraints for organs at risk. Within this context, AI systems seek to overcome limitations in personalisation due to how current models are formulated [6] and answer one or more of the following questions: What dose should be applied to the tumour to achieve a high probability of tumour control? What dose may be present in organs at risk that still would lead to low probability of complications? What additional intervention or treatment modality may improve tumour control, and how should treatment be delivered?

The NTCP-related question is the easiest to answer. AI systems integrate the planned dose in an organ at risk and additional patient-specific characteristics to estimate the probability of adverse radiation effects in healthy tissue. This enables estimation of the patient-specific NTCP [7]. An example use case is the model-based approach for selecting patients to be treated with proton therapy based on the expected reduction in the likelihood of treatment side effects through the use of proton therapy [8]. The involved NTCP models rely on a selection of dosimetric and demographic characteristics as well as accurate recording of clinician- and patient-reported treatment-related toxicities [9, 10]. In recent years, studies have investigated the use of additional data sources such as magnetic resonance imaging of organs at risk [11].

Perhaps surprisingly, the TCP-related questions are harder to answer in research settings using patient data. Patients with a specific diagnosis receive a standard dose prescription that varies little, making it difficult to directly infer the role of dose in tumour control [12]. Research therefore focuses mostly on development and assessment of AI systems that estimate the risk of locoregional tumour recurrence, disease progression, distant metastatic spread and death, and other, similar endpoints [13]. Here, the hypothesis is that patients at a higher risk of adverse tumour-related outcomes may benefit from a change in or addition to the standard treatment, e.g. an escalated dose prescription or the use of specific drugs. Conversely, patients with a very low risk of adverse tumour-related outcomes but with likely treatment side effects may be eligible for a reduction in dose. Despite a large research interest, at the time of writing, very few randomised clinical trials are investigating the interventional use of AI systems for personalising radiotherapy^1^.

## Personalisation during treatment

**Fig. 1:**
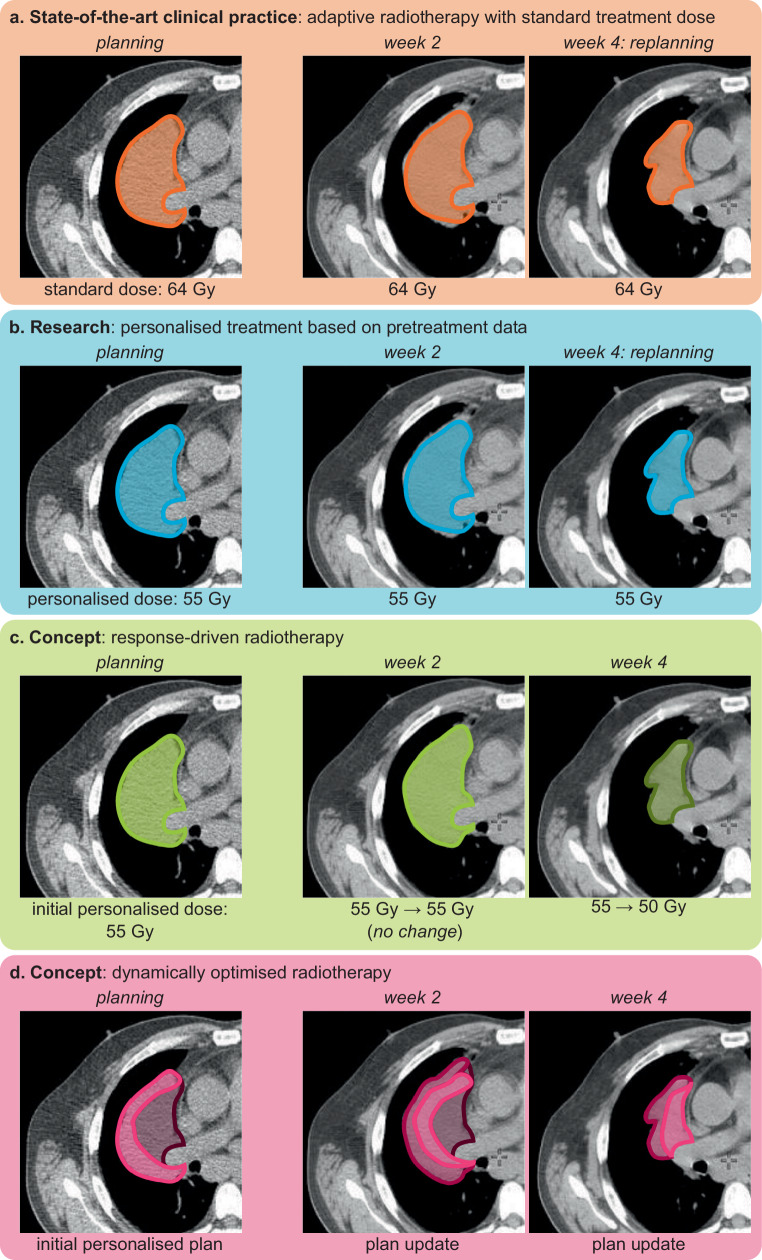
Concepts for personalising radiotherapy using artificial intelligence. The current state of the art does not involve artificial intelligence (AI) systems for personalisation (**a**). A standard treatment dose is prescribed and delivered in an adaptive fashion with replanning. Current research focuses on AI systems that personalise radiotherapy based on pretreatment data (**b**). In this example, the AI system predicts that the prescribed dose may be reduced, while still achieving an effective treatment. The treatment is then delivered in a conventional manner. In response-driven radiotherapy, the initial plan uses predictions of the AI system based on pretreatment data (**c**). As data are acquired during treatment, the AI system uses the observed tumour response to alter the treatment. In this case the good tumour response prompts a reduction in the total dose. Dynamically optimised radiotherapy uses an AI system to dynamically optimise treatment and achieve a curative effect while minimising the dose delivered to normal tissues (**d**). In the presented example, the AI system initially prescribes a higher dose to the outer tumour rim and gradually shifts the high-dose region towards the heart. Both response-driven and dynamically optimised radiotherapy are conceptual, and relatively little research has been done. CT images from Chen et al. [5] under CC BY 4.0 licence

Pretreatment personalisation results in static treatment plans that are easy to integrate into current clinical workflows (Fig. 1). However, many radiotherapy treatment regimens are fractionated. The tumour will change over the course of the treatment and thereafter. Conventional static plans use a series of safety margins to ensure the target receives its prescribed dose [14–16]. However, the use of safety margins to ensure tumour coverage means healthy tissues are more likely to receive higher doses, thus increasing the risk of treatment toxicity. To balance the desire to minimise target margins with the need to treat the whole tumour, technological advances aim to precisely deliver radiation dose to the intended target and are increasingly taking anatomical changes during treatment into account in a process called adaptive radiotherapy [17]. Such changes are typically observed by imaging. AI systems are expected to play an important role in this process, e.g. by enhancing low-dose imaging for detecting anatomical changes [18], through automated dose estimation [19] and by measuring dose delivery [20] among others.

Adaptive radiotherapy makes treatment more precise, which may reduce normal tissue toxicities. Altering the actual radiation dose based on observed tumour and normal tissue responses is also starting to be explored. Conceptually, a differentiated tumour response is expected to be observable during fractionated radiation treatment because of differences in tumour radiosensitivity. Indeed, studies point to improved differentiation of treatment response based on changes observed in imaging obtained during treatment [21, 22]. Data that capture tumour response can then be used to support treatment decisions, e.g. early termination or a dose decrease for patients with tumours that respond well to treatment [23], thus paving the way for response-driven radiotherapy. In response-driven radiotherapy, AI systems help interpret complex longitudinal data such as imaging and provide treatment decision support.

The logical conclusion of treatment personalisation is radiotherapy that is dynamically optimised throughout treatment. Dynamically optimised radiotherapy integrates both pretreatment data, data obtained during treatment and biophysical simulations, to create treatment plans that maximise TCP and minimise NTCP. Such plans, which are continuously adapted and reassessed during treatment, can become substantially different from the current single- or few-dose-level treatment plans. Dynamically optimised radiotherapy may introduce evolutionary principles in radiotherapy with the aim of reducing radiotherapy resistance [24, 25]. Due to the complexity of the procedure, dynamically optimised radiotherapy will likely be driven by AI systems.

## Follow-up

Patients who have undergone radiotherapy are regularly checked to assess treatment success, determine any adverse treatment effects, and to monitor tumour recurrence and progression. The need for additional treatment is also determined. AI systems may assist in all these processes and could potentially be used to provide early indications of potential progress, recurrence or metastasis.

For example, the Response Evaluation Criteria in Solid Tumors (RECIST) is a widely used system for assessing treatment response during follow-up of patients with solid tumours [26]. RECIST defines response categories of the treated lesion: complete response, partial response, progressive disease and stable disease. Determining treatment response using RECIST requires measuring lesion sizes, e.g. from imaging. Multiple studies have investigated using AI systems for RECIST scoring to save time and reduce interobserver variability [27–29].

Pseudoprogression of lesions is sometimes observed after radiotherapy [30] and immunotherapy [31]. Pseudoprogression indicates an increase in lesion size that is transient and disappears during follow-up but would be considered progressive disease at the moment of measurement. Pseudoprogression that is interpreted as true progression may result in unnecessary treatment changes. AI systems may help to differentiate between pseudoprogression and true progression [32].

## Data

AI systems require data. During development, data are used to train an AI system, i.e. it learns patterns in the data that are related to tumour control or the response of healthy tissues. A trained AI system can subsequently make predictions based on data from new patients.

**Fig. 2:**
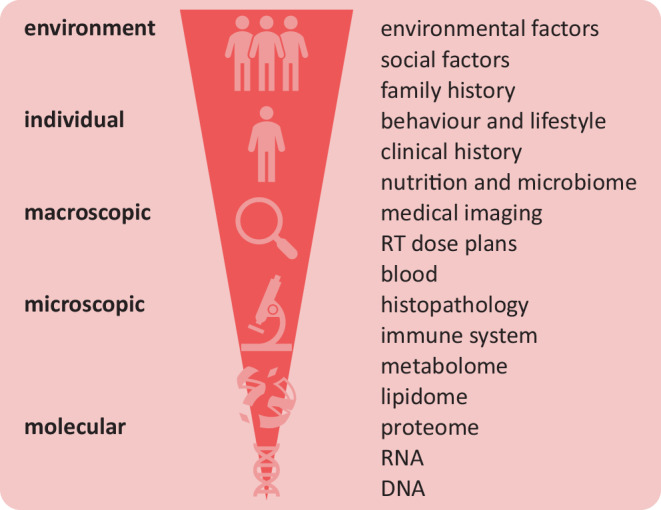
Variety of data available for treatment personalisation. These data range from environmental factors to molecular data

A large variety of data is potentially available for treatment personalisation (Fig. 2). However, acquisition of any data has a cost, both in terms of personnel, equipment and consumables, as well as in terms of patient comfort and wellbeing. Realistically, the patient data available to AI systems are data that are obtained in clinical routine within a relevant timeframe. For example, biomarkers may be determined from a tumour biopsy for diagnostic purposes but are not well suited for response-driven radiotherapy due to both lab time and the need for repeated biopsies. When a relatively complete picture is available, multimodal patient data could, in the future, inform a digital twin to simulate treatment response through both statistical learning—AI techniques—and physics-driven modelling [33].

Some data are commonly available and have been investigated for treatment personalisation. Clinical (e.g. tumour staging) and demographic data (e.g. age and relevant lifestyle choices) are almost always available within the constraints of patient privacy and data protection laws. For various cancer entities, biopsies and other tissue samples provide biomarkers for differential diagnosis [3, 34]. Likewise, diagnostic and treatment planning imaging are readily available. The radiotherapy treatment plan itself is also a data source, describing the expected dose delivered to the tumour and healthy tissues [35].

In contrast to pretreatment data, intra-treatment data sources are considerably more limited, if available at all. Here, AI systems must mostly rely on the already delivered dose and imaging, such as low-dose computed tomography, cone-beam computed tomography or magnetic resonance imaging. Some institutions may collect clinician- or patient-reported outcome measures during treatment, but other data are not routinely obtained. Patient demographics and other patient-level data do not meaningfully change over the timescale of radiotherapy treatment. Biopsy-derived data are not available during treatment due to their invasiveness. Thus, aside from imaging and clinician- or patient-reported outcome measures, the only repeatable and acceptably invasive data derive from blood samples [36–38].

## Challenges

The path to routine use of AI systems for personalising radiotherapy is not trivial [39]. Below we discuss some of these challenges.

### Challenges for realising research concepts

The main challenge in realisation of AI systems for personalising radiotherapy from a research perspective is related to data. Most research currently focuses on pretreatment personalisation of radiotherapy, simply because data are already available. Here, notable data sources are diagnostic and treatment planning imaging and clinical and demographic parameters. For more advanced concepts such as response-driven radiotherapy and dynamically optimised radiotherapy, data are more scarce or absent altogether. This makes assessing and realising these concepts difficult. Research into the response-driven radiotherapy concept is expected to benefit from an increased availability of imaging data during treatment due to an uptake in adaptive radiotherapy in clinical settings. An open question is whether the mostly anatomical information from computed tomography or conventional MRI provides sufficient information to realise this concept or whether functional information (e.g. on tumour oxygenation [40, 41] or immune involvement [42]) is required.

One important aspect of data is heterogeneity. Sources of heterogeneity are, for example, variations in imaging equipment and protocols, variation in data annotation (e.g. tumour segmentation), variation in tissue preparation protocols, variation in data processing, variation in radiotoxicity assessment, etc. Heterogeneity not only applies to input data but also to endpoints. Clinician- or patient-reported outcome measures used as a reference standard for NTCP models are, to a degree, subjective [43]. Likewise, TCP-related events (e.g. tumour recurrence, metastasis, etc.) are dated to the follow-up date at which they are observed but were also present—but unobserved—prior to that date. This results in some uncertainty regarding the exact timing of these events. Data sources that are too heterogeneous hamper the detection of meaningful and generalisable patterns by AI systems, basically drowning any signal in noise. Standardisation and harmonisation efforts seek to limit this heterogeneity [44, 45].

However, some degree of heterogeneity cannot be realistically avoided. AI systems need to generalise to new, unseen data to be clinically translatable. This means that AI systems should be trained with data that contains, or mimics, the heterogeneity encountered in clinical practice. The most straightforward way is by training an AI system on data collected from multiple centres, e.g. through federated learning [46] or local, regional, national and international data repositories [47]. Alternatively, or additionally, if heterogeneity can be characterised, new data can be synthesised or existing data augmented to mimic a heterogeneous dataset [48].

Another issue is that data have an age, i.e. each patient dataset is a snapshot of the clinical practice at the moment it is acquired. Over the years, patient outcomes tend to improve through the availability of new treatment modalities (e.g. consolidation durvalumab in stage III non-small cell lung cancer [49]) and technological improvements [3, 50]. Likewise, data quality and availability tend to improve over time, for example through technological improvements in equipment (e.g. time-of-flight positron-emission tomography [51] or long-read sequencing [52]) and cheaper availability. Data age is especially relevant in the context of TCP-related outcomes such as locoregional control and in late chronic toxicity-related outcomes that may require several years of patient follow-up to detect and collect. By the time a patient dataset becomes available for training AI systems, the dataset is several years old. This may cause a *drift* in performance of AI systems [53], where the performance of an AI system in new data is lower than that in older data that were used to train it. Data age cannot be prevented, but its effect can be reduced by collecting from multiple centres, as mentioned earlier. This not so much shortens the time to availability of data but rather decreases the timeframe required for collecting sufficient data to train an AI system as compared to collection by a single centre, thus allowing for the use of more recent data.

### Challenges to clinical translation

Although the usefulness of treatment personalisation based on AI systems is clear, their clinical translation into the radiotherapy department has not been realised. Since models for treatment personalisation directly intervene in treatments, a high level of evidence for their clinical efficacy is required. Such evidence comes, e.g. from interventional randomised clinical trials [54]. Since such trials are costly, an AI system must offer a clear expected benefit.

Therefore, as a first step, proposed AI systems need to be externally validated. This process is not trivial. Validation requires that a dataset be available that is comparable to the training dataset. It requires that the protocol for preparing and processing these data is known and complete. Then it requires that the AI system itself is available or can be reproduced. Finally, it requires that the protocol for processing the output of the AI system is known and available, where this is relevant. Absence of anything from this process prevents external validation. Because of the reproducibility crisis in science, for which the underlying reasons have not been resolved [55], many proposed AI systems are expected to fail validation. Indeed, this was found in a study in patients with locally advanced rectal cancer where AI systems based on pretreatment imaging proposed in the literature for prognosticating tumour were validated [56].

AI systems for personalised radiotherapy response may also be biassed. Biases result in AI systems that perform better in certain populations than in others and can lead to reinforcing existing structures of inequality [57, 58]. These are also risks for AI systems for response prediction and personalisation in radiation oncology. For one, access to radiotherapy is not equal across the globe [59], which means that data used to train these systems derives predominantly from populations with good access to radiotherapy, with modern equipment and a high quality of care. Even within those populations, access to care and treatment response may be determined by socioeconomic factors [60, 61]. To identify benefits and understand such risks, individual AI systems should undergo an impact assessment [62].

### Challenges to clinical implementation

If an AI system is shown to be clinically meaningful and has received the required certification to operate in a clinical environment, it still needs to be successfully implemented in the clinical workflow [63].

For a radiotherapy department inexperienced in managing AI systems, implementation requires considerable effort. Among other things, the IT infrastructure should be prepared to provide the AI system with its required data and to handle its output. Users should be trained in correct use of the AI system. The AI systems should be monitored and undergo quality assurance [64, 65]. Users should be prepared for occasions when the AI system is temporarily unavailable or no longer supported. Moreover, the use of an AI system may lead to loss of expert skill (*deskilling*) and overconfidence in the output of the AI system (*complacency*), which should be addressed [66]. On the other hand, lack of confidence in an AI system may lead to underutilisation and thus diminished benefits.

In the end, successful implementation of an AI system for treatment personalisation can only be assessed through lasting improved patient outcomes. To realise such outcomes, a radiotherapy department will need to learn how to successfully operate such systems.

## Conclusion

AI systems may help to personalise radiotherapy treatment by prognosticating the tumour and normal tissue response to radiation based on multifaceted and complex patient data. If challenges in translating and implementing AI systems can be overcome, we expect that the first AI systems for personalising radiotherapy will use pretreatment data to adapt the prescribed dose. These systems will then likely be improved upon by integrating the observed tumour response before full dynamic optimisation of treatment plans becomes a reality.

## Footnotes

1 ClinicalTrials.gov; visited January 15, 2024.
